# Serpin E2 promotes breast cancer metastasis by remodeling the tumor matrix and polarizing tumor associated macrophages

**DOI:** 10.18632/oncotarget.12927

**Published:** 2016-10-26

**Authors:** Tatiana Smirnova, Laura Bonapace, Gwen MacDonald, Shunya Kondo, Jeffrey Wyckoff, Hilmar Ebersbach, Bérengère Fayard, Arno Doelemeyer, Marie-May Coissieux, Marinus R. Heideman, Mohamed Bentires-Alj, Nancy E. Hynes

**Affiliations:** ^1^ Friedrich Miescher Institute for Biomedical Research, Basel, Switzerland; ^2^ Koch Institute for Integrated Cancer Research, Massachusetts Institute of Technology, Cambridge, MA, USA; ^3^ Novartis Institute for Biomedical Research, Basel, Switzerland; ^4^ University of Basel, Basel, Switzerland

**Keywords:** serine protease inhibitors, collagen I, tumor associated macrophages, breast cancer, metastasis

## Abstract

The extracellular serine protease inhibitor serpinE2 is overexpressed in breast cancer and has been shown to foster metastatic spread. Here, we investigated the hypothesis that serpinE2 creates tumor-promoting conditions in the tumor microenvironment (TME) by affecting extracellular matrix remodeling. Using two different breast cancer models, we show that blocking serpinE2, either by knock-down (KD) in tumor cells or in response to a serpinE2 binding antibody, decreases metastatic dissemination from primary tumors to the lungs. We demonstrate that in response to serpinE2 KD or antibody treatment there are dramatic changes in the TME. Multiphoton intravital imaging revealed deposition of a dense extracellular collagen I matrix encapsulating serpinE2 KD or antibody-treated tumors. This is accompanied by a reduction in the population of tumor-promoting macrophages, as well as a decrease in chemokine ligand 2, which is known to affect macrophage abundance and polarization. In addition, TIMP-1 secretion is increased, which may directly inhibit matrix metalloproteases critical for collagen degradation in the tumor. In summary, our findings suggest that serpinE2 is required in the extracellular milieu of tumors where it acts in multiple ways to regulate tumor matrix deposition, thereby controlling tumor cell dissemination.

## INTRODUCTION

Proteases and their inhibitors are key physiological regulators of extracellular matrix (ECM) remodeling (reviewed in [1]), a process that contributes to metastasis. The extracellular serine protease inhibitor (serpin) serpinE2, also known as PN-1, is overexpressed in various human cancers, including breast [2] and plays an essential role in malignant progression and metastasis [2–4]. However, the mechanism by which serpinE2 promotes metastasis in breast cancer models remains largely unclear.

Following serpinE2 inhibition of target proteases, the serpin/protease complexes are bound and cleared from the extracellular milieu via low-density lipoprotein receptor-related protein-1 (LRP1), a process that simultaneously eliminates the complexes from the TME and activates LRP1 signaling [5, 6]. We have previously shown that serpinE2 knock-down (KD) in an aggressive breast tumor model blocked metastasis [2]. The goal of this study was to uncover the mechanisms underlying the effect that this serpin has on metastasis. For this we analyzed the extracellular environment of the mammary tumors in response to serpinE2 KD or to treatment with a novel serpinE2 targeted antibody. We show here that targeting serpinE2 via KD or by treatment with the antibody causes a reduction in the population of tumor-promoting macrophages, as well as a decrease in chemokine ligand 2 (CCL2), which is known to stimulate macrophage abundance and polarization [7]. Multiphoton intravital imaging revealed deposition of a dense extracellular collagen I matrix encapsulating serpinE2 targeted tumors. In addition, TIMP-1 secretion is increased, which may directly inhibit several MMPs critical for collagen degradation in the tumor. Our results suggest that serpinE2 is required in the extracellular milieu of tumors to regulate tumor matrix deposition, thereby controlling tumor cell dissemination.

## RESULTS

### SerpinE2 blockade increases the density of collagen I tumor matrix

We have previously shown that the aggressive 4T1 mammary tumor model requires the protease inhibitor serpinE2 in order to disseminate from the primary tumor to distant organs [2]. Moreover, we showed that tumor-derived, and not host-derived serpinE2 is essential for metastasis [2]. We examined a second serpinE2 KD model using human MDA-MB435 metastatic breast cancer cells and generated two KD cell lines. Following injection into mammary fat pads of SCID mice, primary tumor growth was not affected by serpinE2 KD (Figure S1 A-D), similar to what was seen with the 4T1 model (Figure S2A) [2]. However, the serpinE2 KD cell lines showed impaired migration and KD tumors gave rise to significantly fewer lung metastases (Figure S1 A-D).

**Figure 1:**
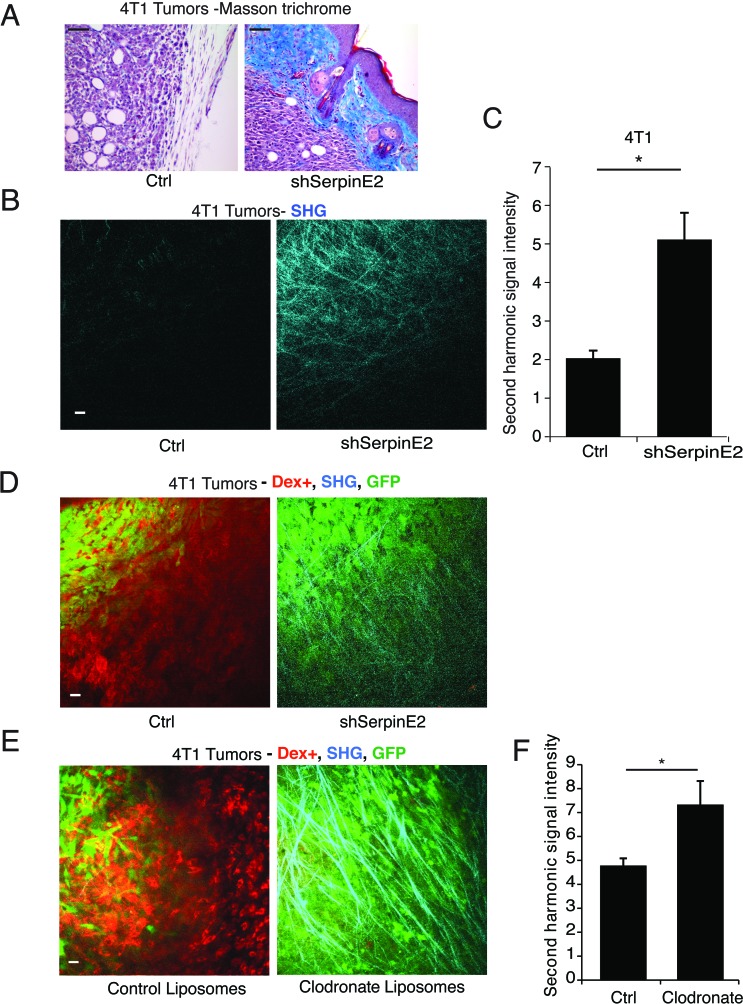
SerpinE2 controls tumor matrix density and matrix-remodeling (**A**) Masson trichrome staining performed on 4T1 Ctrl and shSerpine2 tumors. Representative images show increased collagen encapsulation (blue) of SerpinE2 KD tumors. Scale bar100μm. (**B**-**C**) Intravital imaging using (IVI-MP) of 4T1 Ctrl and shSerpine2 tumors. (B) Representative images show collagen I fibers detected by the SHG signal (cyan) at the surface of 4T1 tumors; scale bar25μm. (C) Average SHG signal intensity was determined per 100μm z-stack; data are acquired from 19-30 separate z-stacks from 3 mice per cell line. *P < 0.00036. **D.** IVI-MP performed on mice bearing GFP-labeled 4T1 control and shSerpinE2 tumors. Representative images show GFP-labeled tumor cells (green), phagocytic dextran positive cells (red); SHG imaging identified collagen I fibers (cyan). Scale bars25μm. (E-F) (**E**) GFP-labeled 4T1 tumor-bearing mice were treated with control liposomes or clodronate-containing liposomes until IVI-MP was performed. Representative images are shown as in (D). (**F**) Quantification of SHG (cyan) signal intensity in 100 μm Z-stacks of tumors in treated animals. Data are mean ± SEM of measurements from 40-61 Z-stacks from at least 3 different tumors for each treatment group. *P < 0.016. All data are mean ± SEM.

Proteases and their inhibitors play important roles in ECM remodeling [1], prompting us to examine the impact of serpinE2 loss on the tumor ECM. In the non-metastasizing 4T1 serpinE2 KD tumors, Masson trichrome staining showed an intense collagen matrix, compared to control metastasizing tumors (Figure 1A). To specifically examine collagen I deposition, which is known to directly influence metastasis [8], intravital imaging by multiphoton microscopy (IVI-MP) was used to visualize collagen I by second harmonic generation (SHG) [9]. Compared to control tumors, IVI-MP imaging of the serpinE2 KD tumors revealed a strong increase in the collagen I-containing matrix (Figure 1B-1C, Figure S2B-C, Movies S1–2), suggesting that this change in the ECM might contribute to decreased metastatic spread of serpinE2 KD tumors.

### SerpinE2 controls matrix-remodeling macrophages

Tumor-associated macrophages (TAMs) have well-known roles in matrix remodeling and degradation [10]. We examined phagocytic cells, which we determined to be mainly macrophages, and not dendritic cells, in these models (Figure S2 G-H and [11]), by injecting Texas red dextran into the blood stream of tumor-bearing mice, before IVI-MP. Compared to controls, serpinE2 KD 4T1 tumors have decreased levels of Texas red dextran positive cells (Figure 1D; Figure S2B-C; Movies S1–2). To test if the alteration in collagen I matrix is due to changes in macrophages, these were depleted with clodronate liposomes. Administration of Texas red dextran before IVI-MP showed that there were few if any dextran positive TAMs remaining in 4T1 tumors after clodronate administration (Figure 1E). Moreover, tumor matrix visualization by SHG revealed restoration of the collagen I matrix when TAMs were eliminated (Figure 1F; Figure S2D-F).

### Serpin E2 loss leads to a decrease in tumor-promoting macrophages and CCL2 levels

**Figure 2:**
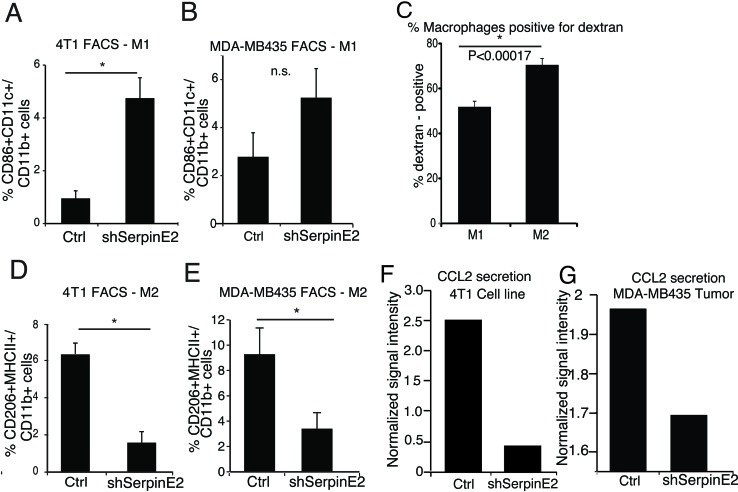
SerpinE2 controls tumor-promoting macrophages and CCL2 levels (**A**-**B**) 4T1 (A) and MDA-MB435 (B) control and shSerpinE2 tumors were harvested and FACS analyses were performed for % of CD86+CD11c+ M1 macrophages in the CD11b+ cell population. (A) *n* = 4 mice per group, * *P* < 0.012; (B) *n* = 3-5 mice per group, *P* < 0.12 (n.s.). (**C**) FACS analyses were performed for the percentage of dextran-positive CD11b+ CD86+CD11c+ M1 and CD206+MHCII+ M2 macrophages from 4T1 control tumor-bearing mice, i.v. injected with Texas Red Dextran 1 hour before dissection. (*n* = 7 mice per group), *P < 0.00017. (**D**-**E**) 4T1 (D) and MDA-MB435 (E) control and shSerpinE2 tumors were harvested and FACS analyses were performed for % of CD206+MHCII+ M2 macrophages in the CD11b+ cell population. (D) *n* = 4 mice per group, * *P* < 0.0022; (E) (*n* = 3-5 mice per group). * *P* < 0.024. All Data are the means ± SEM. (**F**) Cytokine arrays from 4T1 control and shSerpine2 tumor cells in culture. Bars show normalized signal intensity for CCL2. CM from N = 3 plates was used for each cell line. **G.** Cytokine arrays from primary MDA-MB435 control and shSerpine2 tumors. Bars show the normalized signal intensity for CCL2. CM from *N* = 3 tumors was used.

Macrophages acquire distinct phenotypes in response to environmental cues. The classical M1 have anti-tumor properties, while the M2, the major population in the TME, are associated with increased metastasis [12]. Interestingly, the M2-like macrophages were recently shown to be responsible for type I collagen uptake and degradation, *in vivo* [13]. We tested the effects of serpinE2 KD on M1-like and M2-like TAMs, by FACS analyses on M1=CD11b+CD11c+CD86+ cells and M2 =CD11b+MHCII+CD206+ cells (Figure S3A-B gating strategy). In comparison to controls, M1-like TAMS were higher in both 4T1 and MDA-MB435 serpinE2 KD tumors (Figure 2A-2B). The M2-like TAMs, which take up more Texas red dextran than the M1-like TAMs (Figure 2C), were strongly decreased (Figure 2D-2E).

Using a cytokine array analysis on CM from serpinE2 KD tumors and cell lines, we observed that CCL2, which is known to increase macrophage abundance and stimulate their polarization [7], was strongly decreased in response to serpinE2 KD in both models (Figure 2F-2G). Interestingly, an analysis of serpinE2 in the TCGA breast carcinoma dataset using the cBioPortal showed a correlation between the RNA levels of CCL2 and serpinE2 (Figure S3C). In summary, we propose that the drop in CCL2 in the serpinE2-KD tumors contributes to the ensuing decrease in M2-like TAMs and that this has a major role in impeding metastasis.

### Characterization of a serpinE2 blocking antibody

SerpinE2/protease complexes bind LRP1 which clears them from the extracellular milieu; LRP1 signaling is also activated during this process [5, 6]. To test whether the serpinE2 KD phenotypes we uncovered can be recapitulated by interfering with the interaction of serpinE2 complexes with LRP1 in the TME, we produced a serpinE2 specific antibody that recognizes a 12 amino acid peptide, which has been shown to be a structural determinant needed for LRP-mediated internalization of serpin E2/protease complexes and, which can compete for their binding [14–16]. Indeed, using the 4T1 model, we have previously shown that this peptide competes with serpinE2 binding thereby blocking LRP1's signaling activity [2]. To recapitulate this with an antibody, we used recombinant technology to generate a serpinE2 specific antibody targeted to these 12 amino acids, which will be called Ab11 throughout the text (see details in the Methods).

**Figure 3:**
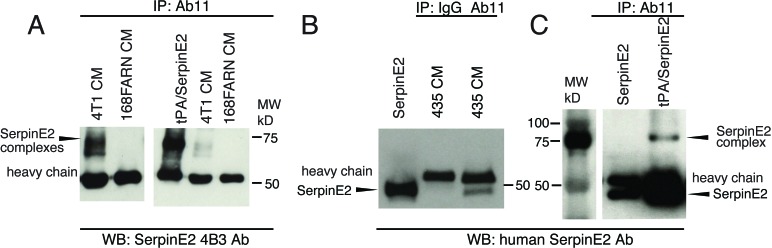
Ab11 immunoprecipates of serpinE2 Conditioned medium (CM) from 4T1 and 168FARN (**A**) or MDA-MB435 (**B**) cultures was incubated overnight with Ab11, then bound serpinE2 was pulled-down with Protein G, and analyzed by western blot with the rodent-specific antibody, 4B3 (A), or a human-specific antibody (B). (A) The right panel shows a short exposure of serpinE2 complexes from 4T1 CM, but none in 168FARN CM (long exposure on the left), as well as an IP of the preformed tPA/serpinE2 complex. (**C**) SFM loaded with purified human serpinE2 or the preformed serpinE2/tPA complex was IP'd with Ab11 as in (A) & (B) and analyzed by western blot with a human serpinE2-specific antibody. The serpinE2 complex and uncomplexed serpinE2 are indicated with arrowheads.

4T1 tumor cells secrete serpinE2 and multiple target proteases [2]. We used conditioned medium (CM) from 4T1 cultures to test the ability of Ab11 to pull-down serpinE2/protease complexes. As a control, CM from cultures of 168FARN cells, which do not produce serpinE2 [2], were also subjected to Ab11 pull-down. The Ab11 bound complexes were then analyzed by westerns for serpinE2 using a rodent serpinE2-specific antibody, 4B3 [17]. Two prominent complexes (Figure 3A left side, shorter exposure, right side), running at the same molecular weight as a preformed rodent serpinE2/tPA complex (Figure 3A right side) were present in the Ab11 pull-down from 4T1 CM, but were not seen in 168FARN CM (Figure 3A). Using CM from cultures of MDA-MB435 cells revealed that Ab11, but not the control isotype matched recombinant antibody (IgG), brought down human serpinE2 (Figure 3B, lanes 3 vs 2), running at the same size as purified serpinE2 (Figure 3B, lane 1). It was not possible to detect serpinE2/protease complexes in CM from these cells, but a complex of preformed human serpinE2/tPA was also recognized by Ab11 (Figure 3C). Taken together, these results show that Ab11 binds to serpinE2 and to serpinE2/protease complexes produced *in vitro* in the extracellular milieu of rodent and human tumor cell lines.

### *In vivo* treatment with Ab11 blocks ERK signaling and decreases metastasis

**Figure 4:**
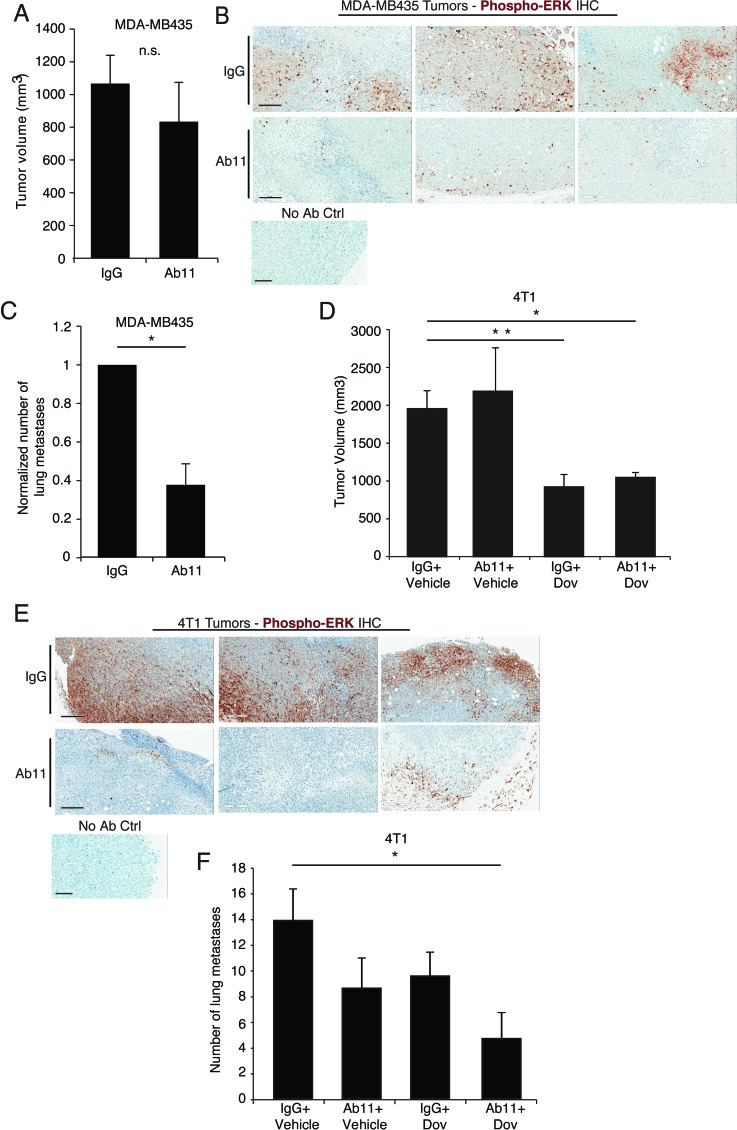
Targeting serpinE2 with Ab11 reduces lung metastasis in breast cancer models (**A**) Tumor volume 7 weeks after orthotopic injection of MDA-MB435 cells into mammary fat pads of SCID mice. Mice were treated with control IgG or Ab11 dosed at 30mg/kg; at least 3 mice per group; n.s. = no significance. (**B**) MDA-MB435 tumors were harvested from mice treated with IgG or Ab11 3 hours before dissection, and were fixed and analyzed by IHC for ERK1/2 phosphorylation (brown staining; representative images from 3 tumors per treatment condition). Small panel shows the “no primary antibody” control staining. (**C**) Spontaneous metastatic potential of tumor-bearing mice from (A) was determined by quantifying visible metastatic nodules on the surface of the lungs. 6-8 animals in the indicated treatment groups. * *P* < 0.0137. (**D**) Tumor volume 23 days after orthotopic injection of 4T1 cells into mammary fat pads of Balb/c mice treated with control IgG +Vehicle, Ab11+Vehicle, control IgG + dovitinib, or Ab11+ dovitinib, as indicated. 6-8 tumors per group. *P < 0.0057, ** *P* < 0.0016. (**E**) 4T1 tumors were harvested from mice treated with either IgG or Ab11 as in (B) and were analyzed by IHC for ERK1/2 phosphorylation (brown staining; representative images from 3 tumors per treatment condition). Small panel shows the “no primary antibody” control staining. (**F**) Spontaneous metastatic potential of tumor-bearing mice from (D) was determined by quantifying visible metastatic nodules on the surface of the lungs. Data are means ± SEM of counts from at least 4 animals carrying tumors from the indicated groups. * *P* < 0.05. Images are from 10X magnification, scale bars = 200 μm. Data are shown as means ± SEM.

Next we tested the *in vivo* effects of Ab11 treatment. Similar to the results obtained with MDA-MB435 serpinE2 KD cells (Figure S1C), blocking serpinE2 with Ab11 did not affect MDA-MB435 tumor growth (Figure 4A). We have previously shown that LRP1 controls ERK pathway activity and that in response to serpinE2 KD ERK activity is strongly decreased [2]. The impact of Ab11 on P-ERK levels, which was examined by IHC carried out on tumor sections, revealed a strong decrease in staining in tumors harvested from Ab11- treated mice, in comparison to IgG control-treated animals (Figure 4B). Importantly, there were significantly fewer lung metastases in the Ab11 treated MDA-MB435 tumor-bearing mice, compared to controls (Figure 4C). Although at this time we have no evidence for Ab11 binding to serpinE2 *in vivo*, the results obtained with the antibody recapitulate those seen with the serpinE2 KD tumors, strongly supporting Ab11's ability to block serpinE2 binding to LRP1.

Turning to the 4T1 model, we have previously shown that fibroblast growth factor receptor (FGFR) is constitutively active in these cells due to autocrine FGF production and that treatment of 4T1 tumor-bearing mice with the FGFR inhibitor dovitinib significantly blocked their growth as well as their metastatic spread [18, 19]. Since the FGFR kinase inhibitor and Ab11 function by different mechanisms, we examined the impact of Ab11 alone or in combination with dovitinib. For this, a sub-optimal dose of dovitinib was used in order to detect potential synergistic effects with Ab11. Treatment with Ab11 had no impact on tumor outgrowth, which is similar to results from serpinE2 KD tumors, however, tumors from dovitinib-treated groups, with or without Ab11, were significantly smaller (Figure 4D). Moreover, an examination of P-ERK levels in the tumors from Ab11-treated mice revealed a strong decrease in activity of the pathway, in comparison to the control tumors (Figure 4E). Finally, the impact of the treatments on tumor spread was examined. Although the observed decrease in the number of lung metastases in the individual treatment groups did not reach significance, there were significantly fewer lung lesions in mice treated with dovitinib + Ab11 (Figure 4F), suggesting that the combination of Ab11 and the FGFR inhibition synergizes to decrease metastasis.

To examine the effect of serpinE2 on extravasation, we used an experimental lung metastasis model. SerpinE2 KD- and control- MDA-MB435 cells were injected in tail veins of mice and lung metastases were quantified, revealing no significant difference between the control and the KD cells (Figure S4A). Using the 4T1 model, we also observed that Ab11 had no effect on extravasation (Figure S4B). These results suggest that in serpinE2 KD tumors, or in response to Ab11 treatment, intravasation of cells might be impaired. To examine this, we looked at tumor cells in the circulation. Blood was collected from mice in the different treatment groups and the number of tumor cell colonies was assessed. In both the MDA-MB435 and 4T1 models, there was a trend for reduced colony number in response to Ab11 treatment (Figure S4C-D), with significance reached in the Ab11+ dovitinib group (Figure S4C). Taken together, the data suggest that fewer cells escape from the primary site when serpinE2 is knocked-down or blocked with Ab11, but once in circulation they form metastases as efficiently as controls.

### SerpinE2 is required for a dense tumor matrix

**Figure 5:**
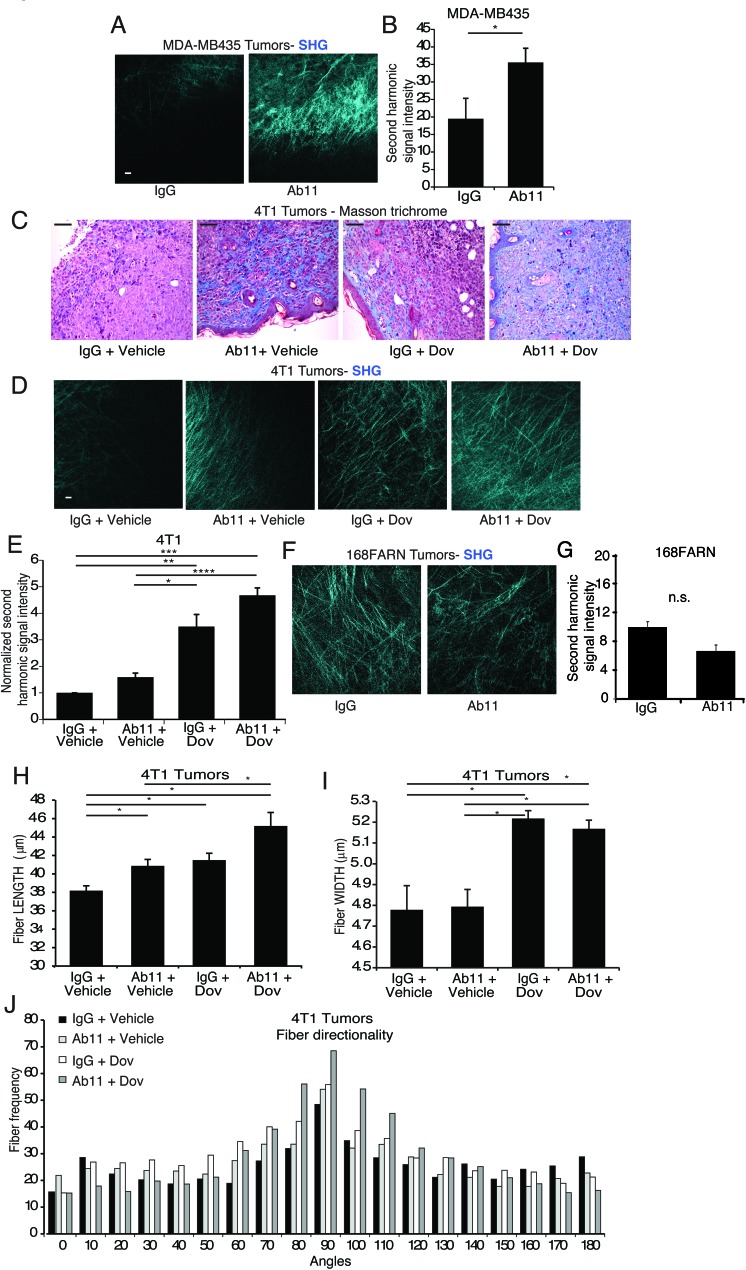
*In vivo* treatment with serpinE2-specific Ab11 increases tumor matrix density (**A**-**B**) IVI-MP of MDA-MB435 mammary tumors from mice treated as indicated, showing representative images of collagen I fibers detected by the SHG signal (cyan) at 0 μm (surface) scale bar25μm. (B) Average SHG signal intensity was determined per 100μm z-stack, from 8-9 separate z-stacks from 3 mice per cell line. *P < 0.031. (**C**) Representative images of Masson trichrome staining, performed on 4T1 tumor sections from the indicated treatment groups, shows collagen encapsulation (blue) resulting from Ab11 and dovitinib treatments. Scale bar100μm. (**D**-**E**) Intravital imaging using IVI-MP, performed on 4T1 mammary tumors in mice treated as indicated, and analyzed as in (A-B). Panels illustrate examples of collagen I fibers detected by the SHG signal (cyan) at 0 μm (surface) for 4T1 tumors (D); scale bar25μm. (E) Average SHG signal intensity was determined per 100μm z-stack; 4-16 separate z-stacks from 3-4 mice per treatment group. *P < 0.0015, **P < 0.0005,*P* < 1.06E-06, ****P < 58E-09. (**F**-**G**) Intravital imaging using IVI-MP, performed on 168FARN mammary tumors in mice treated as indicated, and analyzed as in (A-B). (F) Panels illustrate examples of collagen I fibers detected by the SHG signal (cyan); scale bar25μm. (G) Average SHG signal intensity was determined per 100μm z-stack; 15-16 separate z-stacks from 3 mice per treatment group. (**H**-**J**) Analyses of the structure and organization of the collagen matrix on SHG images of tumors from mice treated as indicated, using the CT-FIRE algorithm for length (H), width (I), and directionality (J) of collagen fibers. N = 15-18 Z-stacks analyzed per treatment condition. *P < 0.05. All data are means ± SEM.

We also examined collagen encapsulation of the tumors in response to Ab11, using IVI-MP to visualize collagen I-containing matrix. In the MDA-MB435 model, Ab11 treatment caused the appearance of a dense matrix on the surface and deeper within the tumors (Figure 5A-5B). Masson trichrome staining showed that 4T1 tumors from both Ab11- and dovitinib- treatment groups had increased fibrillar collagen density, with the combination having the strongest increase (Figure 5C). IVI-MP analysis revealed that Ab11- or dovitinib- treatment alone slightly increased the matrix density, while their combination had the strongest effect (Figure 5D-5E), correlating with the strong block in metastasis. 168FARN tumors, which produce no serpinE2, were also examined. Treatment with Ab11 resulted in no change in matrix density, clearly showing the importance of tumor-produced serpinE2 in the phenotype (Figure 5F-5G).

We also used the curvelet-denoising fiber extraction CT-FIRE algorithm [20] to examine the SHG images of 4T1 tumors (Figure 5H-5J). These analyses revealed that collagen fibers were longer, thicker, and more aligned in the same direction (angle frequency) in tumors from inhibitor-treated animals (Figure 5H-5J). Individually, Ab11 and dovitinib increase fiber length and directionality (Figure 5H & 5J), while dovitinib also blocked degradation since the collagen fibers are thicker (Figure 5I). Thus, the individual treatments have different effects on the collagen matrix, thereby providing an explanation for the striking effect of their combination on blocking metastasis. A cytokine array analysis was also carried out on CM from tumor samples, revealing a strong increase in secretion of the MMP inhibitor TIMP-1, in response to Ab11 treatment and serpinE2 KD (Figure S5A-B).

### Macrophage analysis in Ab11 treated tumors

**Figure 6:**
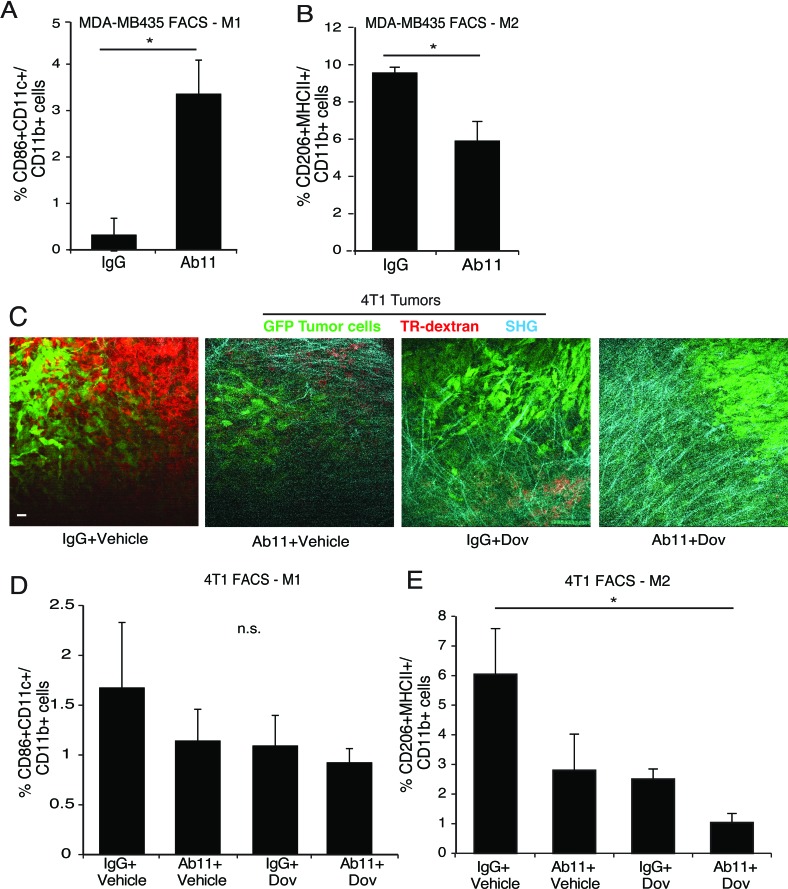
Ab11 treatment leads to a decrease in M2 macrophages and increase in tumor matrix density (**A**-**B**) FACS analysis performed on MDA-MB435 tumors harvested from mice treated as indicated, for % of CD86+CD11c+ M1 macrophages (A) and % of CD206+MHCII+ M2 macrophages (B) in the CD11b+ cell population. (*n* = 3-5 mice per group). *P < 0.007, * *P* < 0.029, respectively. (**C**) Representative images from IVI-MP of 4T1 tumor-bearing mice, treated as indicated, show GFP-labeled tumor cells (green), phagocytic dextran positive cells (red); SHG imaging identified collagen I fibers (cyan). Scale bar25μm. (**D**-**E**) FACS analysis performed on 4T1 tumors from mice treated as indicated, for % of CD86+CD11c+ M1 macrophages (D) and CD206+MHCII+ M2 macrophages (E) in the CD11b+ cell population. (*n* = 3-5 mice per group). * P < 0.024. Data are shown as the means ± SEM.

Finally, we analyzed the TAM populations in tumors from Ab11-treated mice. In the MDA-MB435 model, Ab11 induced a significant increase in M1-like TAMs and a significant decrease in M2-like TAMs (Figure 6A-6B). In the 4T1 model, IVI-MP imaging of phagocytic cells labeled with Texas red dextran was also examined. In comparison to controls with high numbers of dextran-positive cells, mice treated with Ab11, dovitinib, and the combination have strongly decreased Texas red-positive cells (Figure 6C; Figure S6A-D; Movies S3–6). While there were no significant changes in M1-like TAMs (Figure 6D), there was a decrease in M2-like TAMs in response to all treatments, with significance reached in the Ab11 + dovitinib group (Figure 6E). Ab11 also decreased the level of CCL2 secreted from the tumors (Figure S6E).

To further link these results to the matrix phenotype, we examined the SHG images of 4T1 tumors from the clodronate-treated animals (shown in Figure 1E), revealing that collagen fibers were longer, thicker, and more aligned in the same direction (angle frequency) in tumors from the treated animals compared to controls (Figure S6F-H). Thus, with TAM elimination, the tumor matrix becomes less degraded, and more aligned, similar to what is seen with Ab11 treatment.

## DISCUSSION

The influence of the microenvironment on the hallmarks of a tumor are manifold (reviewed in [21]), involving cross-talk between different cell types, as well as interactions with a complex extracellular proteolytic network (reviewed in [1]). We have uncovered a role for serpinE2 in controlling the metastatic potential of mammary tumors, using KD models and a serpinE2 blocking antibody. The effects of inhibiting serpinE2 in tumors are multifaceted: ERK signaling was lowered, CCL2 levels were decreased, a skewing of tumor-promoting M2-like macrophages towards the M1-like phenotype was observed, and the level of the protease inhibitor TIMP1 increased. We speculate that these biochemical and cellular changes all contribute to the deposition of a dense extracellular collagen I matrix, encapsulating the serpinE2-blocked tumors, which inhibits intravasation and tumor spread.

**Figure 7:**
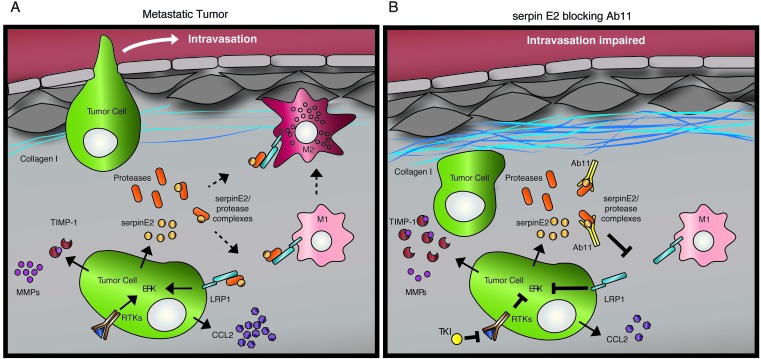
Model depicting the effects of Ab11 treatment on the tumor microenvironment The LRP1 receptor is expressed on tumor cells and on macrophages and we propose that serpinE2 targeting with Ab11 impacts on LRP1 signaling in both cell types. (**A**) In metastatic tumors, serpinE2-LRP1 binding stimulates ERK signaling and secretion of CCL2. Tumor cells display hyperactivation of receptor tyrosine kinases (RTKs); FGFRs, which are active in the 4T1 model, also stimulate ERK pathway activation. We speculate that in macrophages, the combination of high CCL2 levels and serpinE2-activated LRP1 promotes the M2 phenotype. Indeed, there are high levels of phagocytic, Texas-red positive M2 tumor associated macrophages (TAMs) in the metastatic tumors; they are known to be responsible for degrading the matrix during tumor development. (**B**) Ab11 or sepinE2 KD (not drawn in model) lowers ERK pathway activity and the secretion of CCL2, and stimulates TIMP1 secretion. Tyrosine kinase inhibitors (TKIs) block RTK signaling. The matrix-degrading M2 TAMs are decreased in Ab11-treated tumors. Moreover, depleting macrophages with clodronate liposomes results in deposition of a dense collagen matrix, similar to that observed in Ab11-treated tumors. Thus, we propose that the drop in CCL2, which contributes to a decrease in M2 TAMs, as well as blocking serpinE2, which skews the TAMs towards the M1 phenotype, causes the emergence of a dense collagen matrix that inhibits intravasation and metastatic dissemination.

How does serpinE2 affect these processes? A possibility is that LRP1 signaling activity both in the tumor cells, and potentially in cells in the environment, is altered. Based on results showing that activating LRP1 on macrophages promotes the M2 phenotype [22] and that LRP1 activation on some cell types increases CCL2 levels [23, 24], we speculate that macrophages might also be important targets. Since CCL2 is a major player in metastasis [24], directly stimulating tumor cell invasion (reviewed in [25], the decrease in CCL2 observed in response to serpinE2 KD or Ab11 treatment might contribute to blocking tumor cell intravasation, and correlate with M2 polarization [26, 27]. Macrophages have roles in matrix degradation and remodeling [10, 13] and we show that their depletion in tumors causes the appearance of a dense collagen matrix, similar to that observed in the serpinE2 KD and the Ab11-treated tumors. This makes it likely that TAMs are responsible for degrading the matrix during tumor development, and links the decreased M2-like population to a denser collagen matrix. We propose a model whereby SerpinE2 KD/Ab11 treatment both cause a reduction in M2-like TAMs and CCL2 levels, and the emergence of a dense collagen matrix that inhibits metastatic intravasation (Figure 7).

High collagen I density and small matrix pore size present physical barriers to migrating cancer cells [28]. Our work shows that serpinE2 impairment not only causes the emergence of a thick collagen matrix, but also a change in the fiber structure and organization in that they become longer and more aligned. Increased collagen deposition has been shown to correlate with better prognosis in pancreatic cancer patients [29] and, in a pancreatic adenocarcinoma model, collagen I loss resulted in accelerated progression [8]. Considering mammary cancer, increased levels of collagen I in the ECM from parous rats, compared to virgins, has been correlated with decreases in tumor growth and tumor cell invasion [30]. Taken together, we speculate that macrophage skewing towards the M1 phenotype, combined with changes in matrix density, contribute to decreasing intravasation of tumor cells from the primary site.

SerpinE2 is becoming increasingly recognized as having a role in malignant cancer. It was recently shown that 4T1 tumor cells with elevated serpinE2 intravasated more efficiently than controls [31], which is in accord with the data shown here and our previous publication [2]. SerpinE2 overexpression plays an important role in malignant progression and metastasis in different cancer types [3, 4]. In breast cancer, ECM proteomic analyses revealed upregulation of serpinE2 in metastatic cancer cell lines and in tumor tissues [32]. Analysis of serpinE2 in the Cancer Genome Atlas (TCGA) breast carcinoma dataset using cBioPortal [33, 34], revealed that only limited numbers of tumors harbor genetic alterations (6/960), however, 61 show elevated serpinE2 mRNA levels (Fc >1.5). We previously showed that high serpinE2 levels were preferentially found in more malignant grade 3 breast tumors, and in estrogen receptor (ER) negative tumors [2]. Using the TCGA dataset, we found that in tumors harboring high levels of serpinE2 mRNA, there is a negative correlation with *ESR1* mRNA (Figure S7A) and protein (Figure S7B). These results confirm in a much larger dataset that the group of breast tumors with elevated serpinE2 belong to the more aggressive, ER negative sub-type. Interestingly, however, the role of Serpin E2 in cancer might be context specific. It has been shown in a prostate cancer model that high serpinE2 expression inhibits tumor cell invasion and metastasis [35, 36], via its ability to negatively regulate the Hedgehog (Hh) pathway [36]. Decreasing serpinE2 levels led to elevated Hh pathway activity and ensuing increased tumor growth [36]. The negative effect of serpinE2 on Hh pathway activity was first described in the cerebellar granular progenitor cells in the brain [37]. However, in breast cancer models, there is no evidence that serpinE2 regulates the Hh pathway, suggesting that the effects of serpinE2 might be cell type or organ specific.

Clinical efforts to block proteases have been ongoing for years (see for example, [38]), however, increased understanding of how proteases and their inhibitors control the ECM will be needed to assure the best opportunity for therapy. Considering the use of a serpinE2 targeted antibody, we speculate that it would best be used in combination with other targeted agents. The receptor tyrosine kinases are particularly attractive, considering their documented cross-talk with LRP1 [6, 39]. In the work we present here and previously [40], we found that Ab11 and dovitinib inhibit ERK pathway activity, and individually they decrease lung lesions. Their combination, however, is the most proficient in blocking tumor cell intravasation and decreasing lung metastases. In conclusion, we propose that targeting serpinE2 will result in multiple changes in the tumor microenvironment and that combined treatment with a kinase inhibitor might provide additional benefit,

## MATERIALS AND METHODS

### Animal experiments

Animal experiments were performed according to the Swiss guidelines governing animal experimentation and approved by the Swiss veterinary authorities.

### Reagents and antibodies

A monoclonal serpinE2 antibody which recognizes rat and mouse serpinE2 was used for westerns (clone 4B3, production described in [17]). Recombinant tPA was purchased and bound to recombinant serpinE2 as described previously [41]. Human recombinant serpinE2 and tPA were purchased from R&D. Anti-human SerpinE2 Ab (MAB2980) was from R&D systems. The secondary antibody for Western blotting was anti-mouse IgG (LNA931V/AG, GE Healthcare UK), or Veriblot (ab131368, Abcam) for immunoprecipitation experiments. The anti-mouse antibodies for the FACS analyses were all from BioLegend: CD11b (APC-CD11b, cl.M1/70), Ly6C (FITC-LY6C, cl.HK1.4), Ly6G (Percp-Cy5.5-Ly6G, cl.IA8), CD206 (FITC-CD206, cl.C068C2), MHCII (Percp-Cy5.5-I-A/I-E, cl.M5/114.15.2), CD11c (Percp-Cy5.5-CD11c, cl.N418), CD86 (Alexa-fluor 488 CD86, cl.GL-1), CD45 (BV-785); F4/80 (PE, was from eBioscience). Liposomes were purchased from www.ClodronateLiposomes.com.

### Cell lines and cell line generation

The mouse 4T1 and 168FARN cell lines were provided by Dr. J. Yang (UCSD) [42]; the human MDA-MB435 breast cancer cell line [43] was obtained from American Type Culture Collection, Rockville, MD. 4T1 control and shSerpinE2 cell lines were described previously [2]. In that study, two different KD cell lines were characterized (PN-1-shRNA1 clone 1 and PN-1-shRNA2 clone 1) and shown to significantly lower the metastatic ability of 4T1 tumors [2]. For the work described here, we used PN-1-shRNA1 clone 1, referred to as shSerpinE2 KD cells throughout. MDA-MB435 control cells were generated by transduction with a retroviral vector containing shLZ [44]. To downregulate human *SERPINE2*, MDA-MB435 cells were transduced with vectors containing sh15 or sh16 [3]. The shLZ construct was cloned in house [44] into pLKO.1-puro (Sigma). Human serpinE2 shRNA sequences, cloned into pLKO.1-puro, were: CCGGCCTCGTCAACGCAGTGTATTTCTC GAGAAATACACT GCGTT GACGAGGTTTTTG (shSerpinE2, sh15), CCGGGAACACAAAGAAACGCACTTTCTC GAGAAAGTGCG TTTCTTTGTGTTCTTTTTG (shSerpinE2, sh16) [3]. Oligonucleotides were purchased at Microsynth.

For determining secreted serpinE2 levels, equal numbers of cells were plated, and after 12 hours cultures were starved for 24 hours, then CM was harvested, and cell numbers were determined to assure that these were the same in each culture. Secreted serpinE2 levels were determined by a western analysis using a human serpin E2-specific antibody (Figure S1). Both MDA-MB435 KD cell lines exhibited a lower ability to migrate, and MDA-MB435 sh16 (referred to as shSerpinE2 throughout the text) was used for *in vivo* experiments.

For IVI-MP, 4T1 cell lines were transduced with a lentiviral vector encoding GFP (GFP-pWPXL from Sigma). MDA-MB435 cells were transduced with the Luc-2eGFP genes (L2G) [45], in order to express luciferase and GFP proteins. Cultures were selected using puromycin (Sigma) and cell lines were FACS sorted for GFP.

4T1 and MDA-MB435 cells were maintained in Dulbecco's modified Eagle's medium (DMEM, Life Technologies Inc., Rockville, MD, USA) supplemented with 10% fetal calf serum (FCS) (GIBCO Invitrogen) and penicillin/streptomycin solution at 37°C, 5% CO_2_ (Life Technologies, Rockville, MD, USA). Re-authentication was not performed in the last 6 months. For retro- or lenti - viral transductions, cells were infected overnight at 37°C with lentiviral or retroviral particles at a multiplicity of infection of 2, in the presence of polybrene (5μg/ml) (Sigma). The medium was then changed, and the cells were incubated for 24 hours at 37°C.

### Migration assay

Cell migration assays were performed using 8-μm-pore polycarbonate membrane Boyden chambers (Corning Costar Products, Acton, MA) pre-coated with rat-tail collagen I (25 μg ml-1). For this, 100000 cells were seeded in the top chamber in serum-free media. The lower chamber was filled with DMEM containing 0.1%BSA alone or with 5nM EGF (Invitrogen) or 12.5nM HRGβ-1(R&D Systems). After incubation for 16 hours, migrated cells were fixed in 4% formaldehyde and stained with 0.1% crystal violet. Experiments were performed in triplicates; the number of cells per well were counted using a light microscope.

### SerpinE2 Ab11: isolation and characterization

A synthetic mouse based Fab library was cloned in M13 based phagemids (10^13^ phages with 10^10^ different Fabs) and TRIM technology was used to generate diversity in the heavy and light chain CDR3 binding regions [46]. A peptide derived from the mouse serpinE2 (PHENVVVSPHGI), which has been shown to block serpinE2/thrombin binding to LRP1 [14], was used for selection and enrichment of specific Fabs. Three rounds of phage display [47] were performed, with continuously increased washing stringency, to select phagemids with the highest affinity. Pools were sub-cloned and Fabs were produced in bacteria as His-tagged proteins for further characterization. Twelve Fabs, which bound the peptide with low nM affinity, were selected from ELISA screens. After testing for their ability to interact with serpinE2/protease complexes, Fab11 was selected to be cloned into an IgG2a backbone, and was produced in HEK293 cells. We used this antibody, referred to as Ab11, for all experiments. An isotype matched recombinant mouse IgG2a antibody (referred to as IgG) was used as control. Of note, this peptide (PHENVVVSPHGI) does not block the internalization of serpin E2/uPA to LRP1, which has been shown to be mediated by the uPA receptor through a different mechanism [16].

### *In vivo* tumor and metastasis assays

For the 4T1 model, 5x10^5^ cells were injected into the fourth mammary fat pad of 7-week old Balb/c mice (Harlan laboratories, Netherlands); for the MDA-MB435 model, 2x10^6^ cells were injected into the fourth mammary fat pad of 7 week old female SCID mice (Charles River Laboratories, Wilmington, MA, USA). For all experiments, mice were randomly distributed into groups. For all treatments Ab11 and an IgG2a control antibody were used, with or without the FGFR inhibitor dovitinib [48], for the 4T1 model. The antibodies were i.p. injected at 30 mg/kg 2x weekly; 20 mg/kg dovitinib was dosed daily by oral gavage; vehicle control was water. Antibody treatments were started 24 hours post tumor-cell injection; dovitinib treatment was started 7 days post injection. Tumors were measured every 3-4 days and volume was calculated using the following formula: Volume = Height × ((Diameter/2)^2^ × *π*). To determine spontaneous metastasis, 8 weeks (for MDA-MB435) or 23 days (for 4T1) after injection, mice were sacrificed, the lungs were removed and fixed in formalin (MDA-MB435) or Bouin's solution (4T1). The lung sections were stained for vimentin or GFP (MDA-MB435) and quantified, or visible metastases (4T1) were counted.

### Quantitative assessment of metastasis and tumor histology (IHC)

Immunohistochemistrywas done using the Ventana Discovery XT biomarker platform. All lobes of the lung were paraffin-embedded, and 5 μm sections were taken every 100 μm through the entire block and were stained using antibodies against human vimentin (NCL-VIM-V9, Novocastra Leica Biosystems) or GFP (A-11122, Invitrogen Corp). Digital slide image data were generated using a slide scanner (Zeiss MIRAX, version 1.12). Automated quantitative assessment of stained lung tissue areas was performed with Definiens XD software (version XD 2.0, Definiens AG) and an in-house developed image analysis algorithm. Tumor sections were stained using antibodies against Phospho-ERK (Cell Signaling #4370). Masson's trichrome staining of tumor sections was performed according to the instructions provided by the manufacturer (Polysciences, Inc., #25088).

### Intravital imaging by multiphoton microscopy (IVI-MP)

For IVI-MP experiments, GFP-expressing cells were injected into the fourth mammary fat pad of SCID mice and primary tumors were imaged 3-8 weeks post injection [44] on a custom-built microscope [49]. All imaging was performed at 880nm to visualize GFP, Texas Red dextran, and Second Harmonic Generation (SHG) from the tumor surface (0um), until 100um depth. The length, width and orientation of the fibers were determined from SHG images using the CT-Fire algorithm [20].

### Visualization of dextran positive cells and SHG quantification for collagen I by IVI-MP

A 100 m Z-stack at 5-m increments was recorded for each frame starting at the tumor capsule (surface). SHG images of collagen I were quantified for density by determining average pixel intensity; the background (the area of image negative for fibers) was subtracted for all 21 slices of 100 um Z-stacks, using ImageJ. The representative images of SHG, GFP and Texas red dextran are shown in pseudo-color; brightness needed to be adjusted in some representative merged images; these manipulations were not performed for data analysis and quantification. For visualizing macrophages, 100 l of 20 mg/ml 70 kDa Texas Red-dextran (Invitrogen, Molecular Probes) was injected into the tail vein of the mice 30 minutes before surgery and imaging. To visualize the effect of macrophage depletion on the tumor matrix, control or clodronate-containing liposomes (ClodronateLiposomes.com) were prepared according to the manufacturer's guidelines and injected i.p. into SCID mice bearing 4T1-GFP mammary tumors, every 48 hours from days 0-22 of tumor growth. IVI-MP was performed at 880nm.

### Flow cytometry

Tumors were mechanically and enzymatically dissociated using collagenase II (Sigma, Switzerland) and cell suspensions were incubated with 2.5 μg of specific antibodies per 10^6^ cells, or with 2.5 μg mouse IgG isotype control antibody per 10^6^ cells for 30 min at 4°C in the dark, prior to washing and analysis. At least 10^4^ cells per sample were analyzed with a FACScan flow cytometer (Becton Dickinson, Basel, Switzerland). The reference population for gating was SSC^low^CD11b+, thereby excluding granulocytes. M1-like macrophages were identified as SSC^low^CD11b+CD11c+CD86+ cells and M2-like macrophages as SSC^low^CD11b+MHCII+CD206+ cells. For % M1 and M2 macrophages positive for Texas Red Dextran, BD LSRII SORP Analyser was used (Becton Dickinson, Basel, Switzerland).

### Cytokine arrays

4T1 and MDA-MB435 tumors were harvested, and pieces of similar weight were placed in DMEM with 0.1% BSA (SFM) for 2 hours. For cytokine arrays, the CM of 3 individual tumors from each group (volume normalized to tissue weight) was pooled and a cytokine array was carried out using the protocol provided by the manufacturer (R&D Systems, Inc., ARY006). For cytokine analysis *in vitro*, equal numbers of cells were seeded in at least 3 different biological repeats, cultures were starved overnight in SFM, then the same volumes of CM were collected, cell numbers were verified, and the array analysis was performed.

### Intravasation and extravasation assays

To monitor intravasation, blood was drawn and plated as described previously [50]; 12 days later, colonies were counted and normalized to blood volume drawn. To examine extravasation into the lungs, 1x10^5^ 4T1 cells were injected into the lateral tail veins of Balb/c mice that were pre-treated 24 hours prior to injection with Ab11 (30 mg/kg) +/− dovitinib (20 mg/kg), then treated for another 10 days with the agents. 5 × 10^5^ MDA-MB435 cells were injected into the lateral tail veins of 7-week-old female SCID mice. Four weeks (MDA-MB435) or 11 days (4T1) after injection, mice were sacrificed, and the lungs were removed: for MDA-MB453 they were fixed in formalin and sections were stained for GFP; for 4T1 they were fixed in Bouin's solution and visible lesions were counted.

### Statistical analysis

Statistical significance was determined using unpaired, two-tailed Student's *t*-tests, assuming unequal variances and an α-level of 0.05. Differences were considered significant if the *P*-value was < 0.05. For blood burden/intravasation assays, the non-parametric Mann-Whitney or Wilcoxon rank sum test was used. Differences were considered significant if the *P* value was < 0.05.

## SUPPLEMENTARY INFORMATION














