# Supplementary material for: Serpin E2 promotes breast cancer metastasis by remodeling the tumor matrix and polarizing tumor associated macrophages
Source: Oncotarget. 2016 Oct 26;7(50):82289–304. doi: 10.18632/oncotarget.12927 (PMC5347692; doi:10.18632/oncotarget.12927)
Supplement: Supplementary file 1 [file oncotarget-07-82289-s001.pdf]

Serpin E2 promotes breast cancer metastasis by remodeling the tumor matrix and polarizing tumor associated macrophages

Supplementary Information

Movies S.1-2. Visualization of macrophages in 4T1 tumors by IVI-MP from the surface (0um) until 100 um depth in the tumor. Movie shows imaging of 21 slices in a field, Z-slices are 5um apart. Tumor cells are GFP labeled (green), the phagocytic dextran+ cells are red, and SHG imaging is used to identify collagen I fibers (cyan). Scale is 1 um/pixel. **Movie S.1:** 4T1 ctrl. **Movie S.2:** 4T1 shSerpin E2. See Supplementary Video1–2**.**

Movies S.3-6. Visualization of macrophages in 4T1 tumors by IVI-MP from the surface (0um) until 100 um depth in the tumor. Movie shows imaging of 21 slices in a field, Z-slices are 5um apart. Tumor cells are GFP labeled (green), the phagocytic dextran+ cells are red, and SHG imaging is used to identify collagen I fibers (cyan). Scale is 1 um/pixel. **Movie S.3:** 4T1 IgG2a+vehicle. **Movie S.4:** 4T1 Ab11+vehicle. **Movie S.5:** 4T1 IgG2a+dovitinib**. Movie S.6:** 4T1 Ab11+dovitinib**.** See Supplementary Video3–6**.**

Figure S1

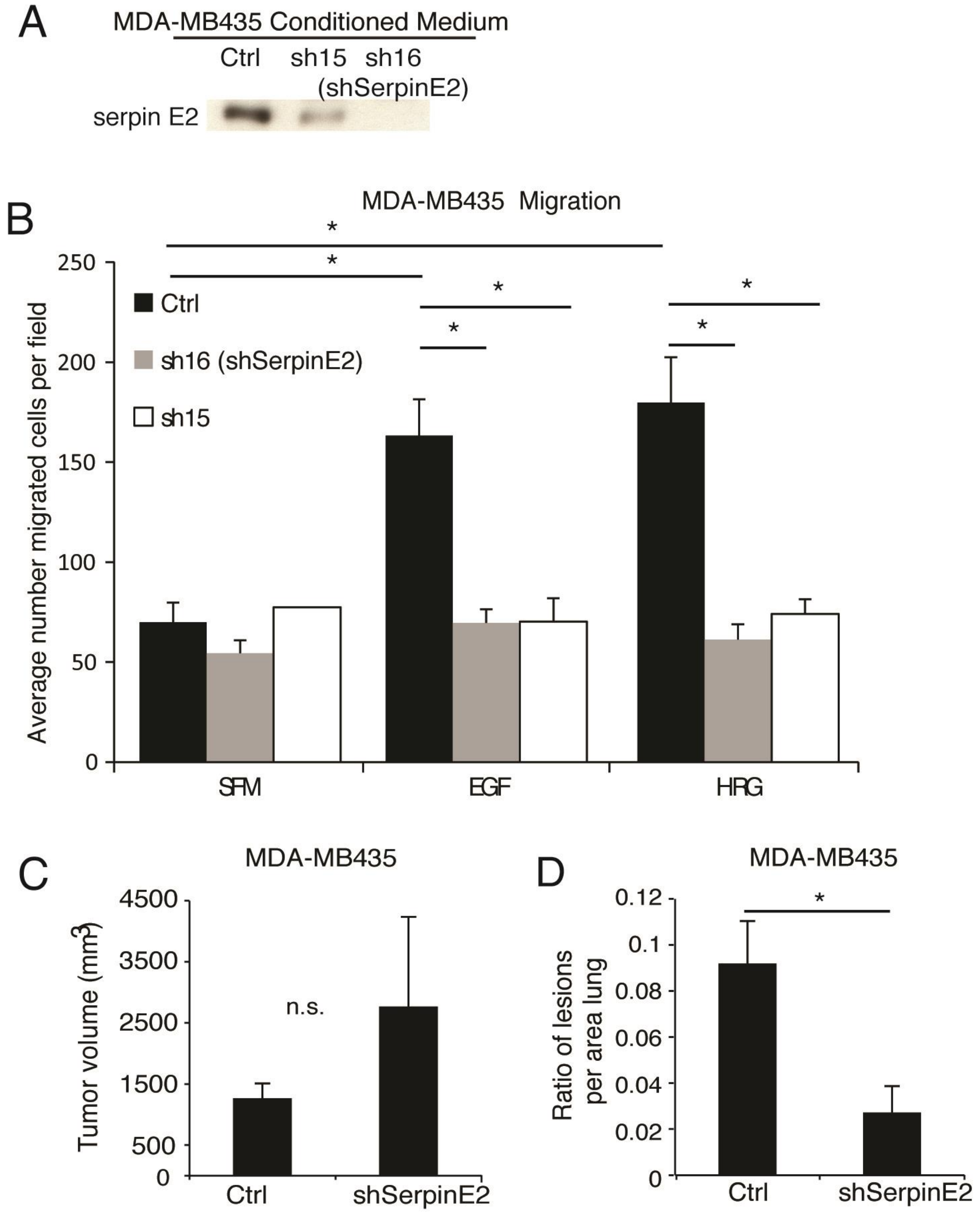

Supplemental Figure S1. SerpinE2 is essential for metastasis of human MDA-MB435 cells

(A) A representative western blot analysis for secreted serpinE2 using a human specific antibody carried out on conditioned medium (CM) of MDA-MB435 shLZ (control) and serpinE2 KD (sh16, sh15) cells plated at the same density. sh16 cells showed the strongest serpinE2 decrease and

were tested for tumor growth and metastatic potential following injection into mammary fat pads of SCID mice (C&D).

(B) Graph shows chemotaxis to serum-free medium (SFM), or SFM with 5nm EGF or 12.5nm HRGβ1 of MDA-MB435 shLZ (Ctrl, black bars), sh16 (gray bars) and sh15 (white bars). Data are mean ± SEM of 4-8 wells in 3 independent experiments. * $P < 0.05$

(C) Tumor volume 8 weeks after injection of MDA-MB435 control (shLZ) or shSerpinE2 cells into mammary fat pads of SCID mice. Data are means ±SEM of at least 4 tumor measurements per group.

(D) Relative number of spontaneous lung metastases per unit lung area, in the animals described in (C). (Data are means ±SEM, n = 3 mice per group). * $P < 0.04$

Figure S2

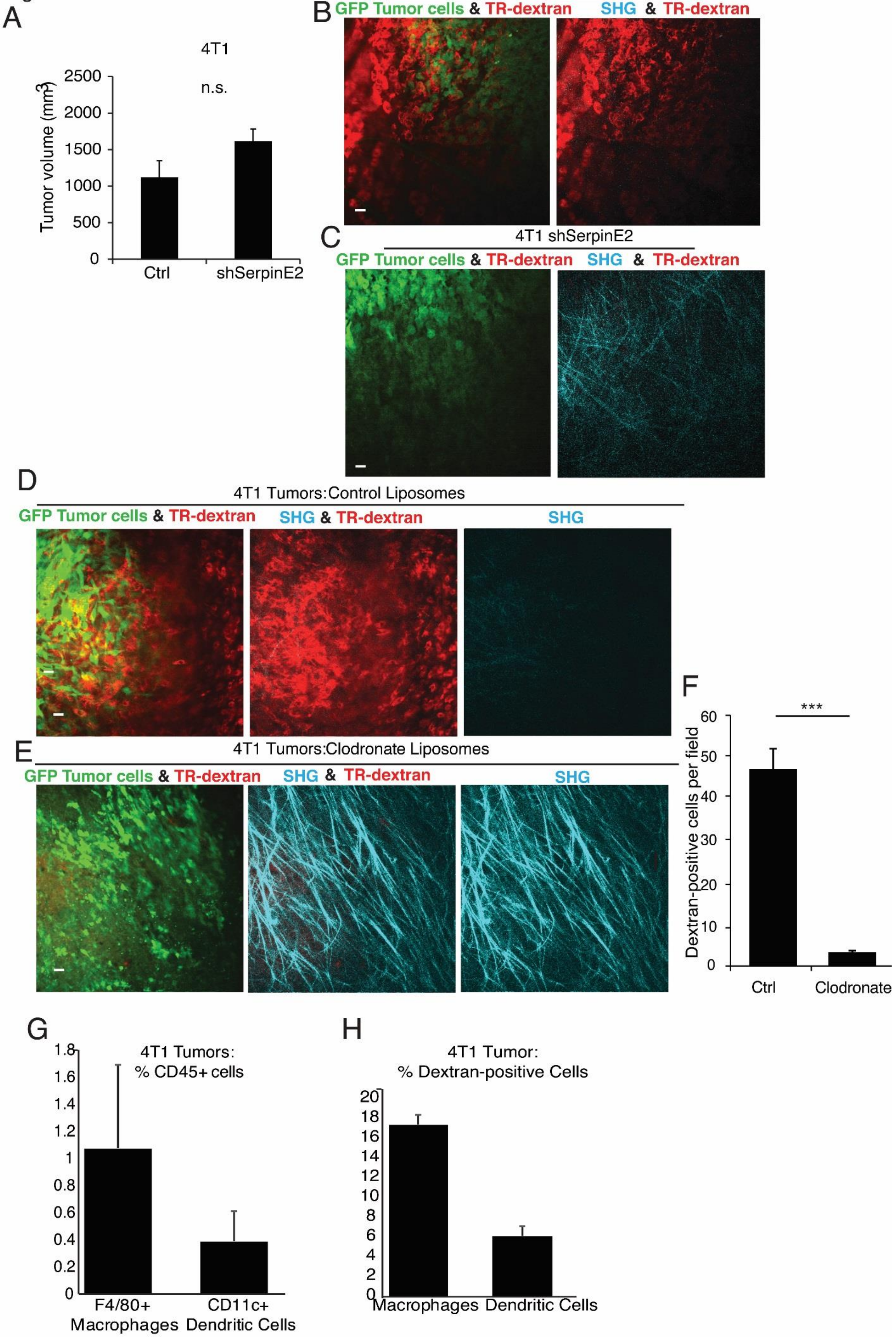
A
4T1
n.s.
Tumor volume (mm3)
2500
2000
1500
1000
500
0
Ctrl
shSerpinE2
4T1 Ctrl
B
GFP Tumor cells & TR-dextran
SHG & TR-dextran
C
4T1 shSerpinE2
GFP Tumor cells & TR-dextran
SHG & TR-dextran
D
4T1 Tumors:Control Liposomes
GFP Tumor cells & TR-dextran
SHG & TR-dextran
SHG
E
4T1 Tumors:Clodronate Liposomes
GFP Tumor cells & TR-dextran
SHG & TR-dextran
SHG
F
***
Dextran-positive cells per field
60
50
40
30
20
10
0
Ctrl
Clodronate
G
4T1 Tumors:
% CD45+ cells
1.8
1.6
1.4
1.2
1
0.8
0.6
0.4
0.2
0
F4/80+
Macrophages
CD11c+
Dendritic Cells
H
4T1 Tumor:
% Dextran-positive Cells
20
18
16
14
12
10
8
6
4
2
0
Macrophages
Dendritic Cells

Supplemental Figure S2. 4T1 tumor characterization, IVI-MP and FACS analyses

(A) Tumor volume 23 days after injection of 4T1 control or shSerpinE2 cells into mammary fat pads of SCID mice. Data are means ± SEM from at least 4 mice

per group. n.s. = not significant.

(B-C) Mice bearing GFP-labeled 4T1 ctrl and shSerpinE2 tumors were injected with 70kDa Texas Red dextran and the mammary tumors were exposed by skin-flap surgery; Z-stacks were acquired by IVI-MP. Representative images show combinations of green and red, and red and blue channels, for better visualization of GFP-labeled tumor cells (green), phagocytic dextran positive cells (red); collagen I fibers (cyan) were identified by SHG imaging. Scale bar=25 µm.

(D-E) GFP-labeled 4T1 tumor-bearing mice were treated with control liposomes or clodronate-containing liposomes until IVI-MP was performed as in (B-C). Representative images show combinations of green and red, and red and blue channels, and blue channels alone, for better visualization of dextran+ cells (red) and collagen I fibers (cyan) in the tumors (green). Scale bar = 25 µm.

(F) Quantification of the number of dextran positive cells in multiple Z-stacks of tumors from animals treated as indicated. Data are mean ± SEM of measurements from 12-56 Z-stacks from at least 3 different tumors for control and clodronate liposome-treated mice. ***$P< 0.0000001$

(G-H) FACS analyses were performed for the percentage of viable CD45+CD11b+Ly6G- F4/80+ macrophages and CD11c+ dendritic cells from 4T1 control tumor-bearing mice (G); FACS analyses were performed for the percentage of dextran-positive cells which are CD45+CD11b+Ly6G-F4/80+ macrophages and CD11c+ dendritic cells (H) from 4T1 control tumor-bearing mice, i.v.

injected with Texas Red Dextran 1 hour before dissection. (Data are mean ± SEM of measurements from *n* = 3-4 mice per group)

Figure S3

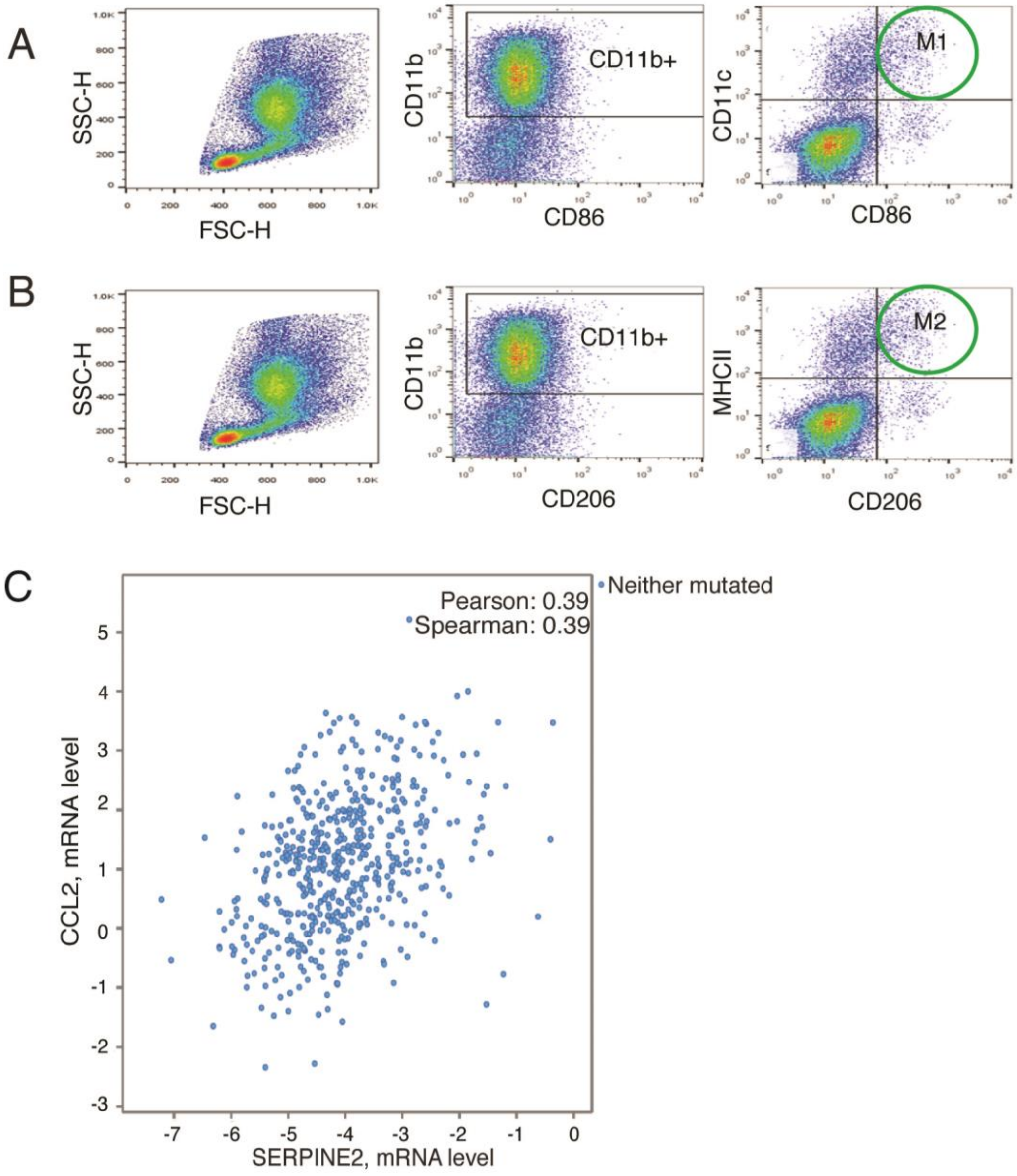

Supplemental Figure S3. FACS gating scheme for M1 and M2 macrophages; CCL2 levels positively correlate with SerpinE2 levels in human tumors

(A-B) Fluorescence-activated cell sorting (FACS) plots showing the gating strategy for identification of CD11b+ CD86+CD11c+ M1 (A) and CD206+MHCII+ M2 (B) tumor-associated macrophages (double-positive populations circled in green)- examples shown from 4T1 tumors.

(C) Analysis of Serpin E2 in the TCGA breast carcinoma dataset using the cBioPortal. Scatterplots show mRNA levels of SerpinE2 (x-axis) versus mRNA levels of CCL2 in 960 human samples.

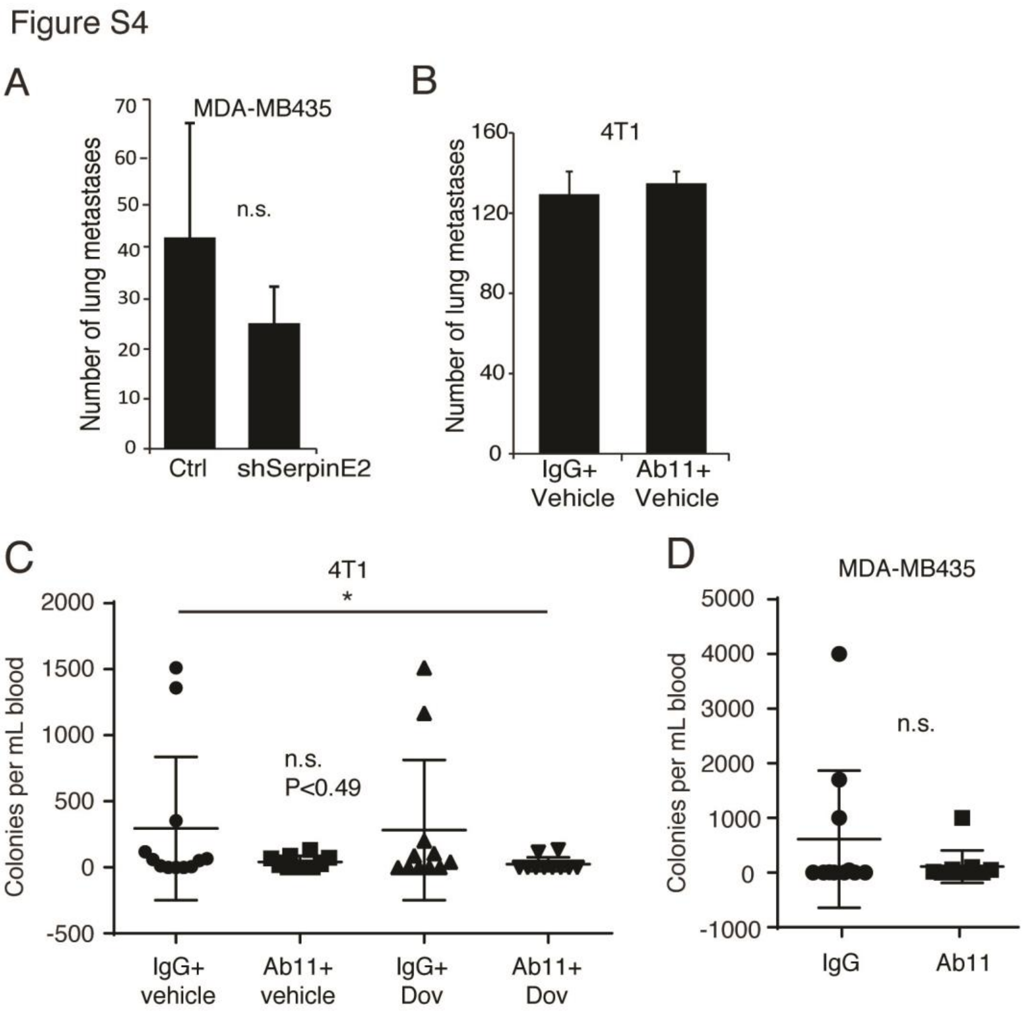

Supplemental Figure S4. Effects of blocking serpinE2 on extravasation and intravasation

(A) Average number of tail vein–derived lung surface metastases ± SEM, 4 weeks after injection of MDA-MB435 shLZ and shSerpin E2 into SCID mice (*n* = 3 mice per group). Data are means ± SEM of 3 animals per cell line.

(B) Average number of tail vein–derived lung surface metastases ± SEM, 11 days after injection of 4T1 cells into Balb/c mice treated as indicated (*n* = 8 mice per group).

(C) Intravasation measured 23 days post-injection of 4T1 cells into mammary fat pads of Balb/c mice in the indicated treatment groups, quantified as the number of tumor cell colonies per ml of blood. 10-12 mice per group. * $P<0.04$ by Mann-Whitney.

(D) Intravasation measured 8 weeks post injection of MDA-MB435 cells into mammary fat pads of SCID mice in the indicated treatment groups, quantified as the number of tumor cell colonies per ml of blood. 5-11 mice per group. n.s. = no significance.

Figure S5

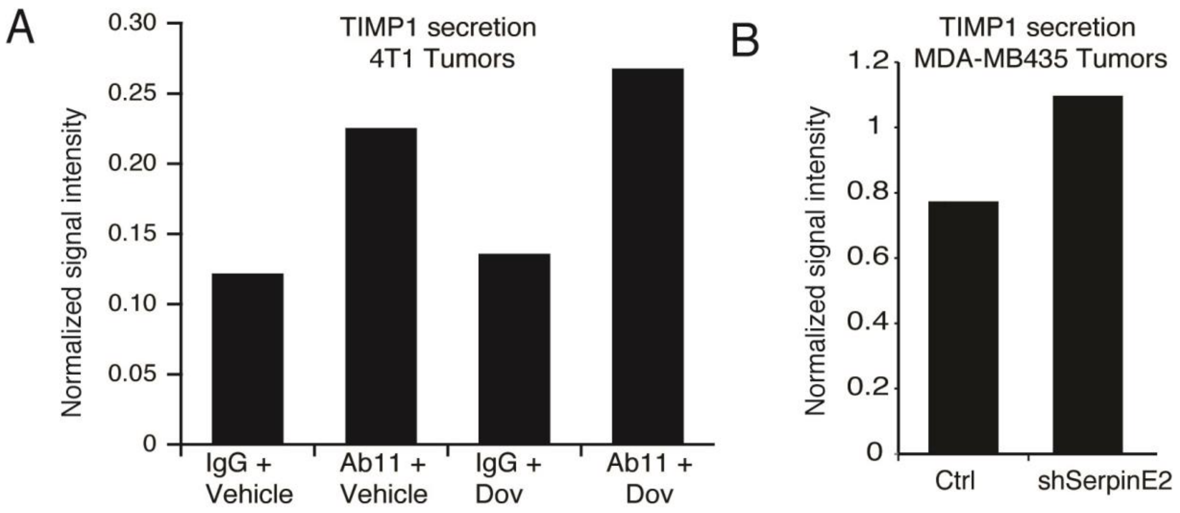

Supplemental Figure S5. Blocking serpinE2 increases TIMP-1 secretion

(A-B) Cytokine arrays from treated 4T1 (A) or MDA-MB435 ctrl and shSerpinE2 (B) tumors. Freshly excised 4T1 tumors were harvested from mice treated as indicated, put into culture and CM was collected 2 hours later. Bars show normalized signal intensity for TIMP-1. CM from N=3 tumors were used for each treatment condition or tumor type.

Figure S6

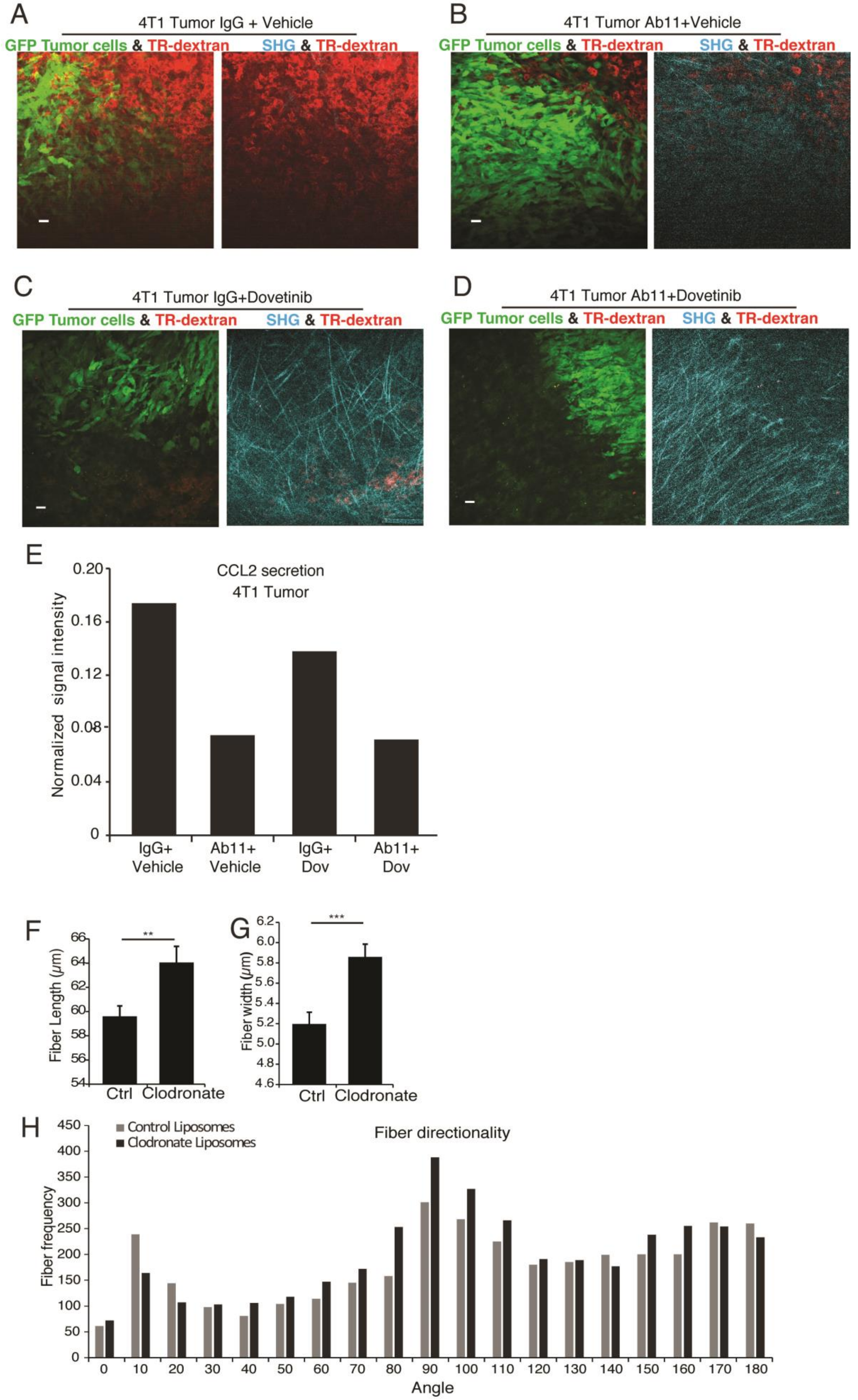
A
4T1 Tumor IgG + Vehicle
GFP Tumor cells & TR-dextran
SHG & TR-dextran
B
4T1 Tumor Ab11+Vehicle
GFP Tumor cells & TR-dextran
SHG & TR-dextran
C
4T1 Tumor IgG+Dovetinib
GFP Tumor cells & TR-dextran
SHG & TR-dextran
D
4T1 Tumor Ab11+Dovetinib
GFP Tumor cells & TR-dextran
SHG & TR-dextran
E
CCL2 secretion
4T1 Tumor
Normalized signal intensity
0.20
0.16
0.12
0.08
0.04
0
IgG+ Vehicle
Ab11+ Vehicle
IgG+ Dov
Ab11+ Dov
F
Fiber Length (μm)
66
64
62
60
58
56
54
**
Ctrl
Clodronate
G
Fiber width (μm)
6.2
6.0
5.8
5.6
5.4
5.2
5.0
4.8
4.6
***
Ctrl
Clodronate
H
Control Liposomes
Clodronate Liposomes
Fiber directionality
Fiber frequency
450
400
350
300
250
200
150
100
50
0
0 10 20 30 40 50 60 70 80 90 100 110 120 130 140 150 160 170 180
Angle

Supplemental Figure S6. Characterization of Ab11-treated tumors; analyses of the effect of TAM depletion on tumor matrix.

(A-D) SCID mice bearing GFP-labeled 4T1 tumors, treated as indicated, were injected with 70kDa Texas Red dextran and the mammary tumors were exposed by skin-flap surgery; Z-stacks were acquired by IVI-MP. Representative images show combinations of green and red, and red and blue channels, for better visualization of GFP-labeled tumor cells (green), and phagocytic dextran positive cells (red); collagen I fibers (cyan) were identified by SHG imaging. Scale bar=25 µm.

(E) Cytokine arrays from primary 4T1 tumors harvested from Balbc/c mice in the indicated treatment groups. Bars show normalized signal intensity for CCL-2. CM from N=3 tumors were used for each treatment condition.

(F-H) Analyses of the structure and organization of the collagen matrix on SHG images of 4T1 tumors from mice treated with control or clodronate-containing liposomes, using the CT-FIRE algorithm for length (F), width (G), and directionality (H) of collagen fibers. N= 15-16 Z-stacks analyzed per treatment condition, N=3 mice per condition. **P< 0.008, ***P< 0.00042.

Figure S7

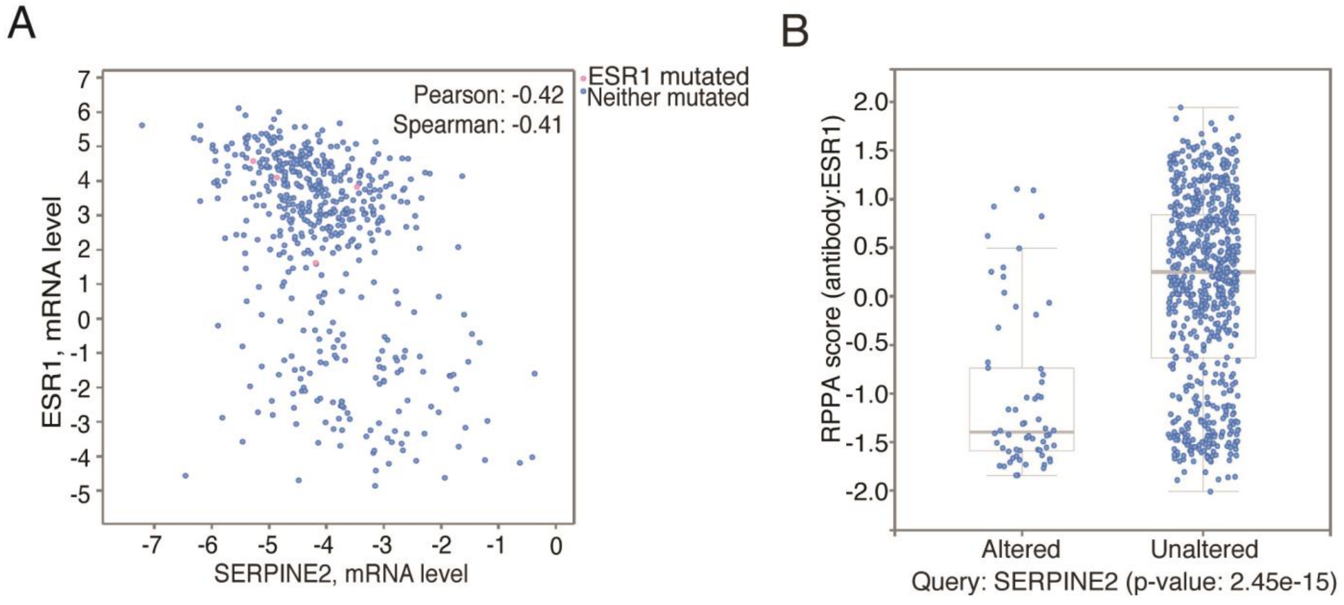

Supplemental Figure S7. SerpinE2 levels negatively correlate with estrogen receptor RNA and protein levels

(A-B) SerpinE2 mRNA levels were analyzed in the Cancer Genome Atlas (TCGA) breast carcinoma dataset using cBioPortal. (A) Scatterplot shows levels of serpinE2 mRNA (x-axis) versus estrogen receptor (ESR1) mRNA (y-axis) in 960 human samples. (B) The X-axis shows groups of patients with altered (upregulated) or unaltered (normal) serpinE2 mRNA levels; Y axis shows estrogen receptor protein levels, determined by reverse phase protein lysate arrays.
